# Prostate cancer radiotherapy and incidental testicular irradiation: Impact on gonadal function

**DOI:** 10.1016/j.ctro.2023.100611

**Published:** 2023-03-08

**Authors:** Jennifer Le Guevelou, Thomas Zilli

**Affiliations:** a Radiation Oncology, Centre Eugène Marquis, Rennes, France; b Faculty of Medicine, University of Geneva, Geneva, Switzerland; c Radiation Oncology, Oncology Institute of Southern Switzerland, EOC, Bellinzona, Switzerland; d Università della Svizzera Italiana, Lugano, Switzerland

**Keywords:** Prostate cancer, Radiotherapy, Testicles, Testosterone levels, Sexual toxicity, Hypogonadism

## Abstract

Incidental testicular irradiation during prostate cancer radiotherapy is rarely documented in literature and the long-term impact on gonadal function largely underreported. Here we present an overview of available data on incidental testicular irradiation and radiation-induced hypogonadism during prostate cancer radiotherapy and discuss future technical developments to minimize testis irradiation.

The long-term impact of androgen suppression has largely been demonstrated in prostate cancer (PCa) patients, with a wide range of hormone-induced side effects, including an increased risk of cardiac events, osteoporosis, metabolic syndrome, and impaired sexual function [1], [2]. While the incidental testicular irradiation in PCa patients has long been known to impact Leydig cells function and testosterone production [3], [4], the extent and clinical impact of testosterone decrease after exclusive radiotherapy remains largely underevaluated. Hereby, we aim to shed light on the existing evidence on incidental testicular irradiation and radiation-induced hypogonadism during PCa radiotherapy and discuss future technical developments to minimize testis irradiation.

The existence of a dose–response relationship between the incidental testicular irradiation and the decrease of the testosterone levels following curative external beam radiotherapy (EBRT) for PCa is far to be well established (Table 1). In patients treated to 68 Gy with standard fractionation using a 3 dimensional conformal radiotherapy (3D-CRT) technique and receiving a mean dose of 2 Gy to the testis, Zagars *et al.* estimated a 9% decrease rate in testosterone levels 3 months after irradiation, but failed to demonstrate a clear correlation between testicular doses and a potential hypogonadal effect [5]. On the other hand, a linear relationship between the dose delivered to the testes and the risk to develop a radiation-induced hypogonadism has been observed by Ishiyama *et al.* [6]: delivering a dose of 76 Gy with an intensity-modulated radiotherapy (IMRT) technique, the mean dose delivered to the testes increased proportionally to 5.3 Gy. A dose of 7 Gy delivered to the testis resulted in a 2-fold reduction in the testosterone levels. After exclusive EBRT [7], 75% of the patients experienced a significant decrease in testosterone level, with a median decrease from baseline to nadir of 30% and a median time to the first decrease of 6.4 months. Up to 45% of the patients experienced biochemical hypogonadism following curative EBRT, with a lower chance of testosterone recovery observed especially in patients with high body mass index, advanced age, and lower testosterone nadir. While in most patients a complete recovery of testosterone levels is observed 12 to 18 months after the EBRT completion [8], [9], up to 40% of the patients are unable to recover their baseline testosterone levels [9].

**Table 1:** Studies evaluating the impact of prostate radiotherapy on testosterone levels and sexual function.

Author	Number of patients	Age, years	RT dose (median)	RT technique	ADT	Follow-up, months	Testicular dose	Hormonal levels	Sexual function
Grigsby *et al.*[20]	59	NS	65–70 Gy	3D-CRT	NR	24	4.5–6 Gy	Similar testosterone levels before and after RT Increased levels of LH and FSH after RT	NR
Tomić *et al.*[21]	31	65	58–71 Gy	3D-CRT	NR	20	Ranging between 1 Gy to > 10 Gy	Lower testosterone levels at 1 week and 3 months after RT Testosterone levels returned to baseline 6–12 months after RT	NR
Zagars *et al.* [5]	85	68	68–76 Gy	3D-CRT	No ADT	3	1.8–2.4 Gy	Lower testosterone levels at 3 months after RT (9%)	NR
Pickles *et al.* [9]	666	72	65 Gy	3D-CRT	3 months ADT (neoadjuvant or adjuvant)	6	2.2 Gy	Lower testosterone levels at 3 months after RT (83% of the baseline level) Recovery to baseline levels for 60% of the patients, within 18 months after RT	NR
Ishiyama *et al.*[6]	39	64	76 Gy	IMRT	No ADT	36	5.3 Gy	Lower testosterone levels at 12, 24, 30, 36 months	NR
Markovina *et al.*[8]	51 (prostate gland: 41; prostate bed: 10)	64	Prostate gland: 73.8 Gy Prostate bed: 64.8 Gy	IMRT	No ADT	24	0.31–2.4 Gy	Lower testosterone levels at 6 months after RT (–33 ng/dL) Testosterone levels returned to baseline 12 months after RT	NR
Pompe *et al.* [7]	248	71	70 Gy	IMRT	No ADT	72	NR	Lower testosterone levels for 75% of the patients after RT (median decrease: 30%)	NR
Oermann *et al.*[19]	26	69	36.25 Gy/5 fx	SBRT (CyberKnife)	No ADT	15	2.1 Gy	Lower testosterone levels for 69% of the patients after RT (median decrease: 3.3 nmol/L) No difference in proportion of patients experiencing hypogonadism (before RT/after RT)	Non-significant decrease in EPIC sexual score: Baseline: 66.7 1-year after RT: 60.1 (p = 0.34)
Yuan *et al.* [10]	636	69	35–40 Gy/5 fx 38 Gy/4 fx	SBRT	No ADT	24	NR	Lower testosterone levels after RT (median decrease: 3–6 months: −13.4%, 7–12 months: −12.2%, 13–18 months: −11.2 % 19–24 months: −5.0%)	No significant decrease in EPIC sexual score on the whole period Significant decrease in EPIC sexual score between 19 and 24 months (10.9 points decline)

*Abbreviations:* 3D-CRT = three-dimensional conformal radiotherapy, ADT = androgen deprivation therapy, EPIC = expanded prostate cancer index composite, SBRT = stereotactic body radiation therapy, RT = radiation therapy, IMRT = intensity-modulated radiotherapy, LH = luteinizing hormone, FSH = follicle stimulating hormone, NR = not reported.

Although the impact of incidental testicular irradiation on gonadal function is determined, the further repercussion on sexual function remains to date speculative. As demonstrated by Yuan *et al.,* despite the testosterone levels 19 months after SBRT returned to baseline and EPIC hormonal scores remained stable, patients showed a 10.9 point decline in the EPIC sexual scores at the 19- to 24-month time period, suggesting a more complex etiology of sexual dysfunction in patients treated with curative radiotherapy [10]. Although this decline in EPIC sexual domain scores was only consistent with a small-sized clinically detectable difference, attention to minimize the incidental testicular irradiation, together with dose optimization to organs at risk like the penile bulb, the pudendal arteries, or the neurovascular bundles [11], [12], [13], [14] should be proposed in an attempt to preserve the erectile function.

The relationship between the incidental testicular dose and the recovery time of testosterone to normal levels has been studied by Pickles *et al.* [15]. Higher doses delivered to the testicles are usually associated with a deeper and more prolonged testosterone nadir and a longer testosterone recovery time. Whether a prolonged testosterone recovery induced by the testicular irradiation is associated with a better biochemical disease control on the long-term remains an unanswered and purely speculative question [16]. Of note, time to testosterone recovery after high-dose radiotherapy combined with androgen deprivation has not been shown to impact clinical failure in the DART01/05 phase III randomized trial [17].

As far as the radiotherapy techniques are concerned, some points merit special considerations. First, while the widespread use of stereotactic body radiation therapy (SBRT) has the potential to decrease the integral dose to the testis compared to standard fractionated regimens, caution to avoid beams directly traversing the testicles and to limit the scattered dose is required when non-coplanar robotic arm devices are used [18]. In a series of 26 patients treated to 36.25 Gy in 5 fractions with a CyberKnife [19], the mean dose delivered to the testis was 2.1 Gy, similar to other series using 3D-CRT [20], [21] or IMRT [6] techniques, but proportionally higher compared to schedules with higher total delivered doses. Nevertheless, in this series, with a median absolute decrease of 3.3 nmol/L at 1-year (–23%), no patient experienced treatment-related hypogonadism after treatment. In a largest series of 636 patients treated with SBRT, Yuan *et al.* were unable to demonstrate a significant change in testosterone levels, even if the subgroup of patients with normal pre-treatment gonadal function was the only one to experience a significant decrease in the testosterone levels at all time periods (approximately 20%) [10]. Second, routine use of image guidance using kilovoltage cone-beam computed tomography (kVCBCT) represents an additional source of incidental irradiation, resulting in up to a 300% increase in testicular dose [22]. While the dosimetric impact of online tumor tracking on the cumulative dose delivered to the testicles has to date not been investigated, non-ionizing tracking systems may represent a non-irradiating alternative for intra-fractional motion control [23]. Similarly, the use of adaptive systems using magnetic resonance-guided radiation therapy (MRgRT) may represent a further step forward in reducing radiation-induced toxicities as demonstrated by the MIRAGE phase III trial [24].

In summary, in the wide spectrum of radiation-induced toxicities, hypogonadism induced by incidental testicular irradiation constitutes an often neglected and underestimated side effect observed in men with PCa treated with curative radiotherapy. Although the relationship with the sexual function and disease control remains to be elucidated, approaches in planning optimization and image guidance trying to limit testes doses are therefore highly encouraged when irradiating curatively PCa patients.

## Declaration of Competing Interest

The authors declare that they have no known competing financial interests or personal relationships that could have appeared to influence the work reported in this paper.
